# Oligonucleotide microarray for the identification of potential mycotoxigenic fungi

**DOI:** 10.1186/1471-2180-10-87

**Published:** 2010-03-23

**Authors:** Sabine Lezar, Eugenia Barros

**Affiliations:** 1 Biosciences, Council for Scientific and Industrial Research (CSIR), PO Box 395, Brummeria, Pretoria, 0001, South Africa

## Abstract

**Background:**

Mycotoxins are secondary metabolites which are produced by numerous fungi and pose a continuous challenge to the safety and quality of food commodities in South Africa. These toxins have toxicologically relevant effects on humans and animals that eat contaminated foods. In this study, a diagnostic DNA microarray was developed for the identification of the most common food-borne fungi, as well as the genes leading to toxin production.

**Results:**

A total of 40 potentially mycotoxigenic fungi isolated from different food commodities, as well as the genes that are involved in the mycotoxin synthetic pathways, were analyzed. For fungal identification, oligonucleotide probes were designed by exploiting the sequence variations of the elongation factor 1-alpha (EF-1 α) coding regions and the internal transcribed spacer (ITS) regions of the rRNA gene cassette. For the detection of fungi able to produce mycotoxins, oligonucleotide probes directed towards genes leading to toxin production from different fungal strains were identified in data available in the public domain. The probes selected for fungal identification and the probes specific for toxin producing genes were spotted onto microarray slides.

**Conclusions:**

The diagnostic microarray developed can be used to identify single pure strains or cultures of potentially mycotoxigenic fungi as well as genes leading to toxin production in both laboratory samples and maize-derived foods offering an interesting potential for microbiological laboratories.

## Background

Mycotoxins are fungal toxins which pose a threat to human, animal and plant health. These toxins can cause acute or chronic toxicity in humans and animals that eat contaminated foods or crops, depending on the quantities produced and consumed [1]. It is estimated that 25% of all food commodities produced on earth are contaminated with mycotoxins due to the fact that fungi develop on these commodities [2]. A study done in South Africa by Rabie et al. [3] showed that mycotoxins such as aflatoxins, beauvericin, deoxynivalenol, moniliformin, trichothecene and zearalenone are contaminants of food commodities. The most known and studied group of mycotoxins in South Africa are fumonisins which have been associated with oesophageal cancer in humans and the cause of leucoencephalomalacia (LEM) in horses, mules and donkeys [4]. It is thus necessary to eliminate or reduce the presence of mycotoxins in the food chain.

An important step in controlling contaminants in the food production chain is by identifying food-borne fungi. The conventional methods used for the detection of fungal contamination are based on phenotypic and physiological characteristics that make use of standard culture and biochemical/serological tests. However, these methods are very time-consuming, laborious and do not detect mycotoxins. Recently, a variety of molecular methods have been used for fungal pathogen identification and for their potential to produce mycotoxins [5]. Molecular methods were used for *Aspergillus *species differentiation using Southern blot hybridization assays [6] and PCR-based restriction fragment length polymorphisms [7]. Most assays that have been developed included PCR-based methods that exploited the highly conserved ribosomal RNA gene sequences for the design of species-specific primers [8] as well as generic PCR detection assays developed for genes involved in the biosynthesis of some mycotoxins [9,10]. Although these assays are an improvement compared to conventional methods, the overall throughput is still limited. Only a limited number of diagnostic regions can be identified for a single organism at a time. If all potentially mycotoxigenic fungi must be included, these assays become laborious and expensive. The use of integrated platforms that combine identification and typing methods for several fungi would facilitate the rapid and accurate identification of possible mycotoxigenic fungi in food commodities.

The microarray technique allows the rapid and parallel characterization of a range of organisms and has the intrinsic ability to perform multiplexed and low-volume biological assays. This technique has been increasingly used for diagnostic purposes as it has the ability to detect more than one parameter at a time [11,12]. Leinberger et al. [13] exploited the polymorphisms of the internal transcribed regions in the ribosomal RNA cassette for the microarray-based detection and identification of *Candida *and *Aspergillus *species. In a similar experiment, DeSantis et al. [14] generated a 62358-probe oligonucleotide of small subunit ribosomal RNA (ssu rRNA) for the detection of 18 different orders of microbes from environmental samples and novel variants exhibiting mutations in their ssu rRNA. Microarrays have also been successfully used to study the expression levels of mycotoxin gene clusters. Schmidt-Heydt and Geisen [15] developed a microarray which contained oligonucleotide probes for the biosynthesis pathways of fumonisin, aflatoxin, ochratoxin, patulin and trichothecene. These studies showed that it is possible to use a range of organisms for hybridization to an array containing many different oligonucleotide probes. The microarray technique is thus analogous to performing many PCR reactions and hybridization reactions at the same time and has the advantage of being versatile [16].

The aim of this study was to develop a diagnostic microarray for the identification of single strains of food-borne fungi that are most prevalent in South African food commodities, and to detect the ability of these fungi to produce mycotoxins in laboratory and food samples. A total of 40 food-borne fungi isolated from different foods that belong to the genera *Alternaria*, *Aspergillus*, *Bipolaris*, *Claviceps*, *Curvularia*, *Diplodia*, *Drechslera*, *Eurotium*, *Fusarium*, *Penicillium *and *Pithomyces*, were used. For fungal discrimination, the polymorphisms of the internal transcribed spacer (ITS) regions and the elongation factor 1- alpha (EF-1 α) gene were exploited for the design of the oligonucleotide probes. The specificity of a probe was increased in some instances by substituting an oligonucleotide with a high affinity DNA analogue known as locked nucleic acid (LNA). A locked nucleic acid nucleotide analogue consists of a 2'-*O*,4'-C methylene bridge and locks the LNA structure into a rigid bicyclic formation and displays unprecedented hybridization affinity towards complementary DNA and RNA [17]. It is most disruptive, and thus gives a better signal, in a centre position. For the detection of fungi that can produce mycotoxins, oligonucleotide probes for the genes leading to mycotoxin production were selected from public databases and included in the oligonucleotide array. The combination of ITS, EF-1 α and mycotoxin genes on the same array was evaluated for the potential of the array to identify the forty fungal isolates and the genes involved in pathways leading to toxin production.

## Results

### Probe design

A 96-probe oligonucleotide microarray was constructed for the simultaneous detection and identification of potentially mycotoxigenic fungi. Probes for the array were designed by exploiting the polymorphisms of the internal transcribed spacer (ITS) regions of the rRNA complex. Amplification of fungal DNA with the universal fungal primers ITS1 and ITS4 and subsequent sequence analysis allowed the differentiation of most of the fungal species studied. Several unique polymorphisms (sequence data can be found in GenBank with accession numbers [GenBank:FJ864706, GenBank:FJ864709, GenBank:FJ864710, GenBank:FJ864708, GenBank:FJ864711, GenBank:FJ864703, GenBank:FJ864704, GenBank:FJ864705, GenBank:FJ864707, and GenBank:FJ864712]) could be identified within the PCR products generated for each fungal species. However, amplification of the *Fusarium *species showed no significant differences between the sequences of the PCR products generated with the ITS primers. Therefore, the elongation factor 1-alpha (EF-1 α) gene was used for the identification of polymorphisms in *Fusarium *species and for the design of unique species- or genus-specific probes. A total of 38 probes could be designed or identified for *Alternaria*, *Aspergillus*, *Penicillium *and *Stenocarpella *species from the ITS regions and 22 probes could be designed or identified for the *Fusarium *species from the EF-1 α gene (Table 1). This probe set was then extended by searching public databases for additional probes for the ITS regions and the EF-1 α gene. No unique probes could be designed for *Drechslera *species, *Eurotium chevalieri*, *Fusarium sambucinum*, *F. semitectum*, *Penicillium funiculosum*, *P. rugulosum *and *Pithomyces chartarum*. The *Fusarium *and *Penicillium *strains share many sequence similarities with the other species used in this study. This rendered the development of species-specific oligonucleotide probes more difficult. For the strains *Pithomyces *and *Eurotium *no unique polymorphisms could be identified that could be used for the design of unique probes.

**Table 1:** Probe sequences and names of species- and toxin- specific genes for different fungal isolates

Probe	Probe sequence (5' → 3')^a^	Probe specificity^b^	PCR annealing temperature (°C) for amplification	Reference (NCBI accession number)
**Internal Transcribed regions**				
AaF AaR	GACCGC**T***TT***C**G**T**GGTATGCA	*Alternaria alternata*	56	This study [GenBank:FJ864712]
AR1	ATCTGCTGCACAGTTGGCT	*Aspergillus carbonarius*	56	This study [GenBank:FJ864707]
AcarF AcarR	TGGC**A**C**C**A**T**TCGTCCTAC CCCGAGGCAGAGATG	*Aspergillus carbonarius*	55	This study [Genbank:FJ864707]
AClF	ATTCGGAAACCUGCTCAGTACG	*Aspergillus clavatus*	58	This study [Genbank:EU515153, EF669942]
AclaF AclaR	GCCGCCGTCTTCGGA CGTGTTG**T**A**C**AA**C**GTTTA	*Aspergillus clavatus*	57	This study [Genbank:EU078633]
ApaF ApaR	GTGTA**C**G**A**G**T**TCCTAGCG GCCCGGGCTGACG	*Aspergillus parasiticus*	55	This study [GenBank:FJ864709]
AVER	CCAAC**G**CA**G**TT**A**CTTCA	*Aspergillus versicolor*	56	This study [GenBank:FJ864703]
ANIG	ACGTTATCCAACCAT	*Aspergillus niger*	55	This study [GenBank:FJ864708]
AnigF AnigR	ATTCGCCGGAGACCCCAACA TGTTGAAAGTTTTAACTGATTGCATT	*Aspergillus niger*	55	This study [GenBank:FJ864708]
EurAF EurAR	TGGCGGCACCATGTC TGGTTAAAAGATTGGTTGCGA	*Eurotium amstelodami*	58	This study [GenBank:FJ864711]
SL24F SL24R	CGGAAGGATCATTACTGAGTG GCCCGCCGAAGCAAC	Penicillium spp., Aspergillus spp.	58	This study [Genbank:AM270353, AM270995, DQ469292, DQ249211]
IT59 ITS60	CGTGTTTATTTACCT ACAGAGCGGTGACA	Penicillium spp.	58	This study [EU7975707.1]
PenCorF PenCorR	GTCCAAACCCTCCCACCCA GTCAGACTTGCAATCTTCAGACTGT	*Penicillium corylophilum*	55	This study [FJ864704]
PenExF PenExR	TTACCGAGTGAGGCCGT GCC**A**GCC**T**GA**C**AGCTACG	*Penicllium expansum*	58	This study [Genbank:FJ861424]
PenFeF PenFeR	CTGAGTGCGGGCCCTCT CGCCGAAGCAACACTGTAAG	*Penicillium fellutanum*	55	This study [Genbank:EF200082]
PenIsF PenIsR	CGAGTGCGGGTTCGACA GGCAACGCGGTAACGGTAG	*Penicilliun islandicum*	57	This study [Genbank:AF455543]
PenItF PenItR	CTCCCACCCGTGTTTATTTATCA TCACTCAGACGACAATCTTCAGG	*Penicillium italicum*	57	This study [Genbank:DQ991463]
ITSF ITSR	CAACTCCAAACCCCTGTGA GCGACGATTACCAGTAACGA	Fusarium spp.	58	[34]
NSA3 NSI1	AAACCCTGTCGTGCTGGGGATA GATTGAATGGCTCGGTGAGG	Internal Transcribed Region	60	[35]
NLB4 NLC2	GGATTCTCACCCTCTATGAC GAGCTGCATTCCCAAACAACTC	Internal Transcribed Region	58	[35]
4F 4R	CAAACGTCGGGTCAGAAGAAGCGAC AGGAACCGTCCCCGCCGACGTTTG	*Styenocarpella maydis*	57	[36]

**Elongation Factor 1-alpha genes**				

EF526F EF1567R	GTCCTGATAGGHCACGT ACGGTCTATACCACCRATCTT	Fusarium spp.	57	This study [Genbank:FJ864705]
FaF FaR	CAAGCATTGTCGCCACTCTC GTTTGGCTCTACCGGGACTG	*Fusarium avenaceum*	60	[37]
FAC-F FAC-R	GGGATATCGGGCCTCA GGGATATCGGCAAGATCG	*Fusarium acuminatum*	57	[38]
CroAF CroAR	CTCAGTGTCCACCGCGTTGCGTA CTCAGTGT**T**CC**G**AATC**T**AATAGTCC	*Fusarium crookwellense*	60	[39]
198F2 198R1	GACAGCAAGATTGACCTTTTGG GACATACTCTACAAGTGCCAA	*Fusarium equiseti*	58	[40]
Fg16NF Fg16NR	ACAGATGACAAGATTCAGGCACA TTCTTTGACATCTGTTCAACCCA	*Fusarium graminearum*	57	[41]
FGDF FGDR	ACATACCACTTGTTGCCTCG CGCCAATCAATTTGAGGAACG	*Fusarium graminearum*	55	This study [Genbank:XM-388987]
FspoF1 LanSpoR1	CGCACA**T**AC**C**C**T**AACTCATC TACAAGAAGAGCGTGGCGATAT	*Fusarium sporotrichioides*	58	This study [Genbank:FJ864706]
61-2F 61-2R	GGCCACT**A**A**T**GA**C**GCGAAAG GTCAGACCAGAGCAATGGGC	*Fusarium subglutinans*	60	[40]
VER1 VER2	CTTCCTGCGATGTTTCTCC AATTGGCCATGGTATTATATATCTA	*Fusarium verticillioides*	56	[42]
53-6F 53-6R	TTACGAGGCGGCGATGGGT GGCCGTTTACCTGGCTTCTT	*Fusarium verticillioides*	60	[40]

**Mycotoxin genes**				

NORTAQ-1	GTCCAAGCAACAGGCCAAGT	Aflatoxin	56	[43]
NORTAQ-2	TCGTGCATGTTGGTGATGGT	Aflatoxin	56	[43]
NORPROBE	TGTCTTGATCGGCGCCCG	Aflatoxin	56	[43]
IDH 2076F IDH 2667R	GCCCATGTGCTCATTACAG TGGGACAATTCCTGAACATGC	Iso-epoxy dehydrogenase	58	[44]
IDH 2195F IDH 2793R	CAATGTGTCGTATGTGCCC ACCTTCAGTCGCTGTTCCTC	Iso-epoxy dehydrogenase	59	[44]
IDH1 IDH2	CAATGTGTCGTACTGTGCCC ACCTTCAGTCGCTGTTCCTC	Iso-epoxy dehydrogenase	59	[45]
Tri7F2 Tri7DON	GTGCGTGGCAATATCTTCTTAGTTA GTGTAATATTGTGCTAATATTGTGC	Deoxynivalenol	58	[46]
Tri13F Tri13DON	CATCATGAGACTTGTKCRAGTTTGG GCTAGATCGATTGTTGCATTGAG	Deoxynivalenol	58	[46]
Fum5F Fum5R	GTCGAGTTGTTGACCACTGC CGTATCGTCAGCATGATGTAGC	Fumonisin	60	[34]
Tri7F Tri7NIV	TGCTGTGGCAATATCTTCTTCTA GGTTCAAGTAACGTTCGACAATA	Nivalenol	58	[46]
Tri13NIVF Tri13R	CCAAATCCGAAAACCGCA TTGAAAGCTCCAATGTCCGTG	Nivalenol	57	[46]
Tri5F Tri5R	AGCGACTACAGGCTTCCCTC AATTCTCCATCTGACCATCCAG	Trichothecenes	58	[47]
TOX5F TOX5R	GCTGCTCATCACTTTGCTCA CTGATCTGGTCACGCTCATC	Trichothecenes	59	[48]

**Positive controls**				

ITS3	GCATCGATGAAGAACGCAGC	Positive hybridization control	55	White et al, 1990
ITS1 ITS4	TCCGTAGGTGAACCTGCG TCCTCCGCTTATTGATATGC	Positive hybridization control	55	White et al, 1990

^a ^Locked nucleic acids (LNAs) that were used to increase the specificity of a probe are in bold and underlined.

^b ^Fungal names should be treated as sensu stricto.

The public databases were also used to identify oligonucleotide probes specific for genes leading to toxin production. The probes selected for the biosynthesis genes leading to the regulation of fumonisins, aflatoxin, nivalenol, deoxynivalenol and tricothecenes were 18 - 22 nucleotides in length. A total of 23 toxin-specific probes were identified and spotted onto the array. The oligonucleotide sequences and their specificities are shown in Table 1. Most probes used for the final array construction were oligonucleotide probes identified in public databases as the probe sequences were diverse and minimal cross-hybridization was obtained. Some sequence data is available upon request.

### Optimization of labeling and hybridization conditions

**Figure 1:**
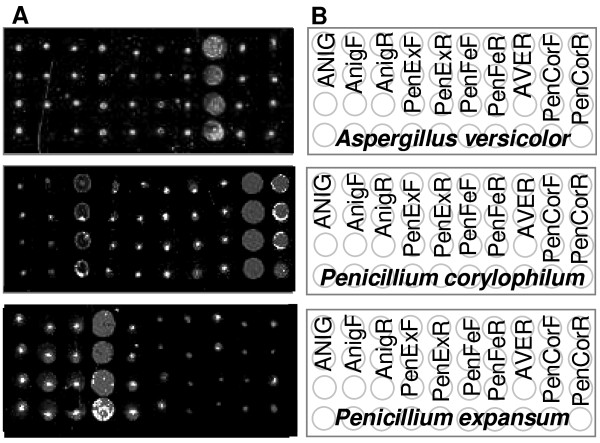
**Sections of fluorescent images showing DNA hybridized to the array**. Sections of fluorescent images after hybridization of target DNA to the diagnostic array. **A**. (Top) Hybridization profile of *Aspergillus versicolor*; (Middle) *Penicillium corylophilum*; (Bottom) *P. expansum*. **B**. The arrangement of a few oligonucleotide probes within the indicated fields of a section of the array. Oligonucleotide probe names were used to indicate the field. Each column represents four replicates of the same spot.

**Figure 2:**
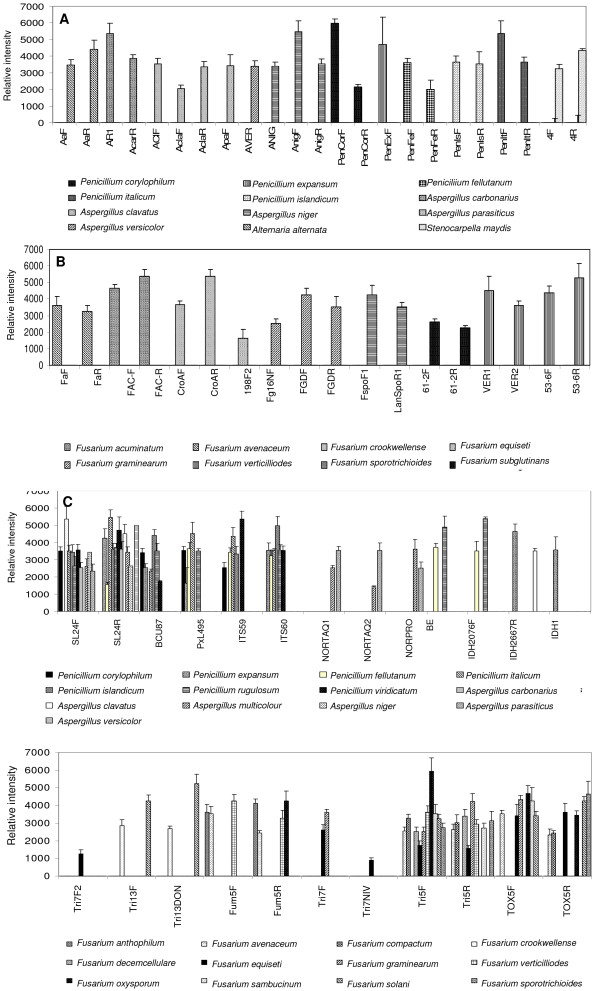
**Relative intensities of hybridized DNA**. Relative intensities after hybridization of labeled target DNA to the array. Each experiment was done in triplicate and the medians and their standard deviations were calculated for each spot on the array. Only positive hybridization results are shown. **A**. Relative intensities of fungal strains hybridizing to probes designed from the internal transcribed (ITS) regions of *Alternaria*, *Aspergillus*, *Penicillium *and *Stenocarpella *species. **B**. Relative intensities of *Fusarium *species hybridizing to probes designed from the elongation factor (EF). **C**. Relative intensities of *Alternaria*, *Aspergillus*, *Penicillium *and *Stenocarpella *species hybridizing to their relevant mycotoxin genes. **D**. Relative intensities of *Fusarium *species hybridizing to their relevant mycotoxin genes.

To avoid amplification bias and to get a more uniform genetic locus representation, targets were labeled using a random approach that does not involve amplification. All labeled target DNA positively hybridized to the array (Figure 1) showing fluorescent net signal intensities ranging from 2000 to 6000 intensity units demonstrating efficient hybridization of the target DNA. The hybridization conditions were further tested to get the optimal discrimination of target species and genes leading to toxin production without having unspecific signal intensities by determining the optimal PCR annealing temperature for fungal DNA using the probes in Table 1. *Aspergillus clavatus *and *A. versicolor *were used for this purpose as they showed cross-hybridization to other species-specific probes in the initial experiment. This was expected as the ITS region of both species are very similar. An increase in hybridization temperature from 42°C to 53°C showed that there is nearly no cross-hybridization between these two species and there was no decrease in net signal intensity (results not shown). Although the ITS sequences are quite similar for both fungal species, high hybridization efficiencies were obtained with net signal intensities of about 2000 signal units for *A. clavatus *and of about 3500 signal units for *A. versicolor *(Figure 2A). In general, it was also observed that the optimal probe annealing temperatures for PCR amplifications was about 5°C higher than the optimal probe hybridization temperature (results not shown). The probes and their optimal annealing temperatures are listed in Table 1.

### Specificity and functionality of the microarray

The specificity of the array was tested by using the forty precharacterized fungal isolates listed in Table 2. The hybridization of fungal isolate to the array gave insight into the affinity of test probes for their correct target and the effect of multiple versus single diagnostic probes/species. The hybridization of each fungal isolates for 16 - 24 hours at 53°C resulted in different hybridization patterns for the different fungal strains (Figure 1) with relative intensities indicating the level of hybridization of each target to the probe (Figure 2). Thirty-two test samples showed high affinity for their probes producing a best match result. It was possible to positively identify the test organisms with at least one probe due to the presence of multiple diagnostic probes with fluorescent net signal intensities ranging from 2992 to 6000 intensity units. SNR values obtained from the relative intensities of hybridized DNA indicated in the graph, gave a clear indication whether a spot was present (SNR>/= 3.0) or absent (SNR<3.0). Weak cross-hybridization was observed for *Aspergillus clavatus *and *A. niger*, but these fungal isolates could be positively identified due to the multiple probes on the array. Although the multiple probes per species used for the array construction showed big differences in hybridization efficiencies with some probes showing no hybridization, at least one oligonucleotide showed high hybridization efficiency for most of the fungal isolates tested and could be used for species- or toxin-specific gene identification. Eight species could not be positively identified as they did not reveal specific hybrization patterns (Table 3).

**Table 2:** Fungal cultures used in this study, their potential mycotoxins and the host of the fungus

No.	Fungus^a^	Isolate identified by conventional method	Mycotoxins produced	Host
1	*Alternaria alternata*	PPRI 2917	Tenuazonic acid	Citrus
2	*Aspergillus carbonarius*	PPRI 5382	Ochratoxin A	Grapes
3	*Aspergillus clavatus*	PPRI 6026	Cytochalacin E, patulin	Unknown
4	*Aspergillus multicolor*	PPRI 5548	Aflatoxin	*Palystes castaneus*
5	*Aspergillus niger*	PPRI 5017	Ochratoxin A	Onion seed
6	*Aspergillus parasiticus*	PPRI 5990	Aflatoxin	*Arachis hypogaea*
7	*Aspergillus versicolor*	PPRI 3645	Sterigmatocystin	*Melianthus comosus *seed
8	*Bipolaris sorokiniana*	PPRI 6180	Sterigmatocystin	Panicum hay
9	*Claviceps purpurea*	PPRI 7268	Ergot alkaloids	*Zea mays*
10	*Curvularia lunata*	PPRI 6099	7-epi-brefeldin A	Lawn
11	*Drechslera *spp	PPRI 4853	Cytochalasin E	*Zea mays*
12	*Diplodia maydis (Stenocarpella maydis)*	PPRI 1048	Diplosporin	*Zea mays*
13	*Eurotium amstelodami*	PPRI 4851	Sterigmatocystin	*Aerospora *sp
14	*Eurotium chevalieri*	PPRI 6368	Sterigmatocystin	Amitide tablets
15	*Fusarium acuminatum*	PPRI 7731	Diacetoxyscirpenol, moniliformin	Unknown
16	*Fusarium anthophilum*	PPRI 7744	Fumonisins	Unknown
17	*Fusarium avenaceum*	PPRI 7685	Fumonisins, moniliformin	Unknown
18	*Fusarium compactum*	PPRI 7734	Fumonisins	Unknown
19	*Fusarium crookwellense*	PPRI 6748	Deoxynivalenol, Nivalenol	Apple soil
20	*Fusarium decemcellulare*	PPRI 5623	Fumonisins	Kapok roots
21	*Fusarium equiseti*	PPRI 7735	Moniliformin, Nivalenol	Unknown
22	*Fusarium globosum*	PPRI 7753	Beauvericin, fumonisins, moniliformin	Unknown
23	*Fusarium graminearum*	PPRI 1199	Deoxynivalenol, Nivalenol, Trichothecin, Zeralenone	Unknown
24	*Fusarium moniliforme\verticllioides*	PPRI 6525	Fumonisins	*Zea mays*
25	*Fusarium oxysporum*	PPRI 7730	Fumonisins, moniliformin	Unknown
26	*Fusarium rugulosum*	PPRI 5412	Fumonisins	Honey
27	*Fusarium sambucinum*	PPRI 4562	Fumonisins	*Setaria uncrossata*
28	*Fusarium semitectum*	PPRI 6174	Fumonisins, moniliformin	Beetle
29	*Fusarium solani*	PPRI 5014	Moniliformin, Nivalenol	Coffee
30	*Fusarium sporotrichioides*	PPRI 7680	T-2 toxin	Unknown
31	*Fusarium subglutinans*	PPRI 7666	Beauvericin, fumonisins, moniliformin	Unknown
32	*Penicillium corylophilum*	PPRI 5993	Cyclopiazonic acid	Imported food
33	*Penicillium expansum*	PPRI 5944	Patulin	Heniocha
34	*Penicillium fellutanum*	PPRI 6340	Patulin	Wheat straw
35	*Penicillium funiculosum*	PPRI3634	Patulin	Peanuts
36	*Penicillium italicum*	PPRI 5925	Patulin	Plum
37	*Penicillium islandicum*	PPRI 4632	5,6, dihydro-4-methoxy-2H-pyran-2-one	*Zea mays*
38	*Penicillium rugulosum*	PPRI 5412	Patulin	Honey
39	*Penicillium viridicatum*	PPRI 4788	Patulin	*Glycine max *seed
40	*Pithomyces chartarum*	PPRI 1254	Sporidesmin	*Debris*

^a^Fungal cultures were identified by the Agricultural Research Council, Pretoria, South Africa, with standard laboratory procedures.

**Table 3:** Fungal species identified (+) or not identified (-) with diagnostic chip developed

No.	Fungus	Array results		Blind samples	
		Genus	Species	Genus	Species
1	*Alternaria alternata*	+	+	+	+
2	*Aspergillus carbonarius*	+	+		
3	*Aspergillus clavatus*	+	+		
4	*Aspergillus multicolor*	+	+		
5	*Aspergillus niger*	+	+	+	+
6	*Aspergillus parasiticus*	+	+		
7	*Aspergillus versicolor*	+	+	+	+
8	*Bipolaris sorokiniana*	+	+		
9	*Claviceps purpurea*	+	+		
10	*Curvularia lunata*	+	+		
11	*Drechslera *spp	-	-		
12	*Diplodia maydis (Stenocarpella maydis)*	+	+		
13	*Eurotium amstelodami*	+	+		
14	*Eurotium chevalieri*	-	-		
15	*Fusarium acuminatum*	+	+	+	+
16	*Fusarium anthophilum*	-	-		
17	*Fusarium avenaceum*	+	+		
18	*Fusarium compactum*	+	+		
19	*Fusarium crookwellense*	+	+		
20	*Fusarium decemcellulare*	+	+		
21	*Fusarium equiseti*	+	+		
22	*Fusarium globosum*	+	+		
23	*Fusarium graminearum*	+	+	+	+
24	*Fusarium moniliforme\verticllioides*	+	+		
25	*Fusarium oxysporum*	+	+		
26	*Fusarium rugulosum*	+	+		
27	*Fusarium sambucinum*	-	-	+	-
28	*Fusarium semitectum*	-	-		
29	*Fusarium solani*	+	+		
30	*Fusarium sporotrichioides*	+	+		
31	*Fusarium subglutinans*	+	+		
32	*Penicillium corylophilum*	+	+		
33	*Penicillium expansum*	+	+	+	+
34	*Penicillium fellutanum*	+	+		
35	*Penicillium funiculosum*	-	-		
36	*Penicillium italicum*	+	+		
37	*Penicillium islandicum*	+	+	+	+
38	*Penicillium rugulosum*	**-**	**-**		
39	*Penicillium viridicatum*	+	+		
40	*Pithomyces chartarum*	-	-		

Note: Eight blind samples were used to evaluate the array and could be identified to either genus- or species-level.

The three controls (ITS1, ITS3, ITS4) which were specific for universal fungal sequences served as internal standards to ensure that the parameters (labelling and hybridization) were similar across experiments. A similar intensity of controls across slides indicated that the relative signal intensities of probes are also similar across slides. Further, some probes in this study were modified to contain locked nucleic acids (LNAs) in at least two selected single nucleotide polymorphisms (SNP) sites per fragment. SNP's were found to be most effective, and thus gave better signal, if they were in a centre position. A probe with multiple polymorphisms along the probe length, regardless of position or modification at the polymorphic site, showed less cross-hybridization (results not shown) which is consistent with the data obtained by You et al. [18].

The functionality of the microarray was tested by hybridizing precharacterized fungal isolates to the array. Twenty-five fungal isolates were characterized for the presence of mycotoxin genes by growing them at 25°C for 1 week, extracting genomic DNA and PCR-amplified the DNA of each individual fungal isolate using the toxin-specific oligonucleotide probes that were used for array construction. Different species showed different amplifications of toxin-producing genes (Table 4). These results indicated which fungal isolates have the potential to produce mycotoxins and hybridized to probes specific for genes leading to toxin production on the array. The amplicons obtained were consistent with the signal intensities obtained when samples were hybridized to the array (Figure 2C-D). The microarray chip developed was also tested for its ability to detect genes leading to mycotoxin production without any knowledge about the identity of the fungal isolate. In this study, *Fusarium anthophilum *was used to test this approach as no species-specific probes were present on the slide. The hybridization of this fungus to the *fum5*F and *fum5*R probes (Figure 2C-D) indicated that the fungus is able to produce fumonisins confirming that mycotoxin-producing genes can be detected. It should be noted that the presence of a gene in the genome does not mean that a gene is transcribed and expressed.

**Table 4:** Fungal species screened and scored for for presence (+) or absence (-) of mycotoxin genes with PCR

Fungal species	Mycotoxin gene specific primers									
	*fum5*	*tri5*	*tri7*	*tri13*	IDH1	IDH2	IDH2076	IDH2667	IDH2195	IDH2793
*Fusarium acuminatum*	-	+	-	-	-	-	-	-	-	-
*F. anthophilum*	+	+	-	-	-	-	-	-	-	-
*F. avenaceum*	+	+	-	-	-	-	-	-	-	-
*F. compactum*	-	+	-	-	-	-	-	-	-	-
*F. crookwellense*	-	-	-	+	-	-	-	-	-	-
*F. decemcellulare*	-	+	-	-	-	-	-	-	-	-
*F. equiseti*	-	+	+	-	-	-	-	-	-	-
*F. globosum*	-	-	-	-	-	-	-	-	-	-
*F. graminearum*	-	+	+	-	-	-	-	-	-	-
*F. oxysporum*	+	+	-	-	-	-	-	-	-	-
*F. rugulosum*	-	-	-	-	-	-	-	-	-	-
*F. sambucinum*	-	+	-	-	-	-	-	-	-	-
*F. semitectum*	-	-	-	-	-	-	-	-	-	-
*F. solani*	-	+	-	+	-	-	-	-	-	-
*F. sporotrichioides*	-	+	-	-	-	-	-	-	-	-
*F. subglutinans*	-	-	-	-	-	-	-	-	-	-
*F. verticillioides*	+	+	-	-	-	-	-	-	-	-
*Penicillium corylophylum*	-	-	-	-	-	-	-	-	-	-
*P. expansum*	-	-	-	-	+	+	-	-	-	-
*P. fellutanum*	-	-	-	-	-	-	+	+	-	-
*P. italicum*	-	-	-	-	-	-	-	-	-	-
*P. funiculosum*	-	-	-	-	-	-	-	-	-	-
*P. islandicum*	-	-	-	-	-	-	+	+	-	-
*P. rugulosum*	-	-	-	-	-	-	+	+	-	-
*P. viridicatum*	-	-	-	-	-	-	-	-	-	-

### Validation of the array

The performance and reproducibility of the array was tested starting from independently extracted fungal DNA from eight blind fungal samples that were hybridized to the array. Binary scores obtained from the array were compared to the binary scores from replicate experiments. Repeatability of the binary scores obtained from the hybridizations from replicate experiments of the same fungi were on average 95%. The results obtained were also compared in each case to the identity obtained for the same culture grown by standard laboratory procedures and to the correlation of the PCR product amplified from the same sample with the positively identified oligonucleotide probes. The same procedure was followed for the mycotoxin biosynthesis genes. The identities of the amplicons and the identities of the fungi obtained by standard methods showed that the array was able to identify the fungi and mycotoxin genes correctly; seven of the eight fungal isolates could be identified up to the species level (Table 3). *Fusarium sambucinum *could not be identified to species level due to the absence of species-specific signals. In all cases the genes leading to mycotoxin production could be identified.

## Discussion

The identification and detection of fungi has become increasingly dependent on molecular characterization. Methods such as Southern blot hybridization assays, restriction fragment length polymorphism analysis and PCR-based assays exploiting the internal transcribed spacer (ITS) and elongation factor 1-alpha (EF-1 α) regions are all effective for the detection and identification of food-borne fungi. However, all these methods can identify only a single organism at a time. Suitable detection methods, anticipating mycotoxin risks, are needed to ensure a safe food production chain and eliminate the risk factors. Oligonucleotide microarrays have a high multiplexing capacity and have proved to be an efficient approach to overcome these limitations. This technology offers an identification process based on sequence confirmation through hybridization [16] and has the ability to analyze many samples simultaneously.

In this study, a microarray chip was developed for the identification of fungal isolates, as well as biosynthesis genes of the most important mycotoxins that can threaten the safety and quality of food products especially those derived from maize. For the design of genus- and species-specific probes the ITS regions of the rRNA gene cassette were exploited. These coding regions show a high degree of variation [19] and analysis of the fungal ITS alignments revealed significant differences among the different fungi. However, analysis of the ITS regions of *Fusarium *species showed that they have similar sequences which could have cross hybridized on the array, making it non-specific. Kane et al. [20] found that in 50mer oligonucleotide arrays, cross-hybridization occurred between fragments of relatively low sequence similarity. The highly repetitive DNA content of plant genomes resulted in cross-hybridization of DNA fragments to printed-probe DNA and the overall spot intensity of many probes was increased. Therefore, the EF regions were used for the design of species-specific probes for *Fusarium *species. For some probes with similar sequences the chances of cross hybridization were minimized by substituting a single oligonucleotide in the probe sequence using a high affinity DNA analogue known as locked nucleic acid (LNA) at three specific points to increase the specificity and the T_m _of a probe. The LNAs were inserted at a single nucleotide polymorphism (SNP) site for improved performance of the probe. Letowski et al. [21] found that probes containing polymorphisms toward the centre of the probe showed a higher discrimination power. If LNAs are to be included then they must be inserted in a triplicate series around the centre of the probe. Further, G-T mismatch sites must be avoided and should preferably be inserted at sites where adenine is the identity of the base [18].

Cross hybridization has also been reported in several microarray-based species detection studies where single regions were used for identification. Anthony et al. [22] found that in oligonucleotide arrays, cross-hybridization occured between *Listeria *species and it was necessary to include additional probes to the array. In a similar study done by Volokhov et al [23], *E. coli *and *Salmonell*a isolates produced indistinguishable hybridization profiles when single probes were used. However, they showed that multiple probes improve the sensitivity of the array when compared with the single diagnostic probes that could be unsuitable for a group of closely related organisms. In this study, the probes spotted onto the array were a mixture of single and multiple probes for each species that were either genus-, species-specific or specific for genes leading to toxin production. When multiple probe sequences were used the discriminatory power of the array increased as a sample hybridized to at least one probe of the multiple probes on the array. In addition, probes for the array construction were designed around a Tm of 56°C so that all probes would hybridize under similar conditions. Considering that microarray experiments are non-equilibrium measurements, it is desirable that microarray probes exhibit uniform thermodynamic properties, which many probe design tools aim to achieve by demanding a narrow distribution of the probe melting temperature T_m _[24]. The fungi hybridizing to the diagnostic array may, however, represent a taxon or haplotype that was not included in the array design. In some of the species complexes included in this study several haplotypes of ITS1 and/or TEF1a genes may be found suggesting that probes my fail to detect some of the haplotypes. The cross hybridization that was observed between *A. clavatus *and *A. niger *indicates that more strains need to be studied and additional probes still need to be designed to discriminate between these two species. This also applies to the eight fungal species that could not be identified to species level.

The random labeling strategy used in this study was applied to diminish secondary structures [25] and to have an efficient target. Previous studies suggested that amplification products of large samples resulted in poor hybridization and target PCR amplification resulted in amplification bias [26]. Although high levels of amplification are desirable for PCR assays, this feature is less critical for microarrays as only limited probe is available on the array surface [16]. As target genomic DNA was not a limiting resource in this study, a random approach that omits the target amplification step prior to DNA hybridization proved to be efficient for the sensitive detection of fungi. This approach ensured that there is an equal amount of target sequences available for dye coupling and thus their representation on the array was balanced. This makes the microarray an attractive tool for single strain fungal infections compared to morphological identification. Zheng et al [27] identified the three fungal pathogens, *Candida*, *Cryptococcus neoformans *and *Aspergillus *directly from 27 clinical specimens using a microarray. However the ability of the present microarray to reliably detect mixed infections and single copy genes such as TEF1a was not established. It is also likely that in a sample containing multiple fungi, the fast-growing fungi are extracted in greater concentrations than the slow-growing fungi making the identification of all the fungi present in the sample not possible.

The microarray developed was also evaluated for its ability to detect genes leading to toxin production without prior knowledge of the fungus that produced it. Determination of toxin producing genes is often of a greater concern than the identification of the exact fungal species. Although our understanding of the biosynthesis of mycotoxins is incomplete several genes have been identified. Often more than one gene plays a key role in the biosynthetic pathway and it is important to include as many genes as possible on the microarray chip for proper identification of toxin-producing fungi. In this study, two unknown *Fusarium *species that show no species-specific signals on the array were identified to genus level by their positive hybridization to the mycotoxin-producing genes *tri5*, *tri13 *and *fum5*F and *fum5*R, respectively. However, based only on the hybridization signal it was not possible to predict whether the respective mycotoxin was produced. This could have been achieved if cDNA would have been used as a target in the array hybridization where differentially expression of mycotoxin genes would have indicated mycotoxin production. Schmidt-Heydt and Geisen [15] used RNA to detect the activation of gene clusters under conditions conducive for the biosynthesis of trichothecenes, fumonisin, ochratoxin, aflotoxin and patulin. However, they found that the biosynthesis of secondary metabolites, like mycotoxins, is dependent on environmental conditions like substrate, pH, temperature and water activity [28] and thus mycotoxins are not always expressed.

## Conclusions

With the multiplexing capacity as one of the important features of microarrays, the method developed in the present study can be used to detect more than one parameter at a time, namely fungal species and genes involved in pathways leading to toxin production. A total of 32 fungi could be identified and their potential to produce mycotoxins could be determined. This study describes the omission of the target amplification step of target DNA prior to hybridization in a DNA-based microarray experiment. The results indicated that the random labeling technique could provide enough labeled target DNA for the direct detection of a single fungal infection from infected maize kernels using the microarray In the long term, the developed microarray chip could be used to hybridize DNA and cDNA labeled with different Cy dyes for the simultaneous detection of fungal identity and toxin involved genes. The genomic DNA would determine the fungal identity and the cDNA would determine whether genes for mycotoxin biosynthesis are expressed. The cDNA approach can also be useful to determine which gene clusters are expressed under conditions conducive for the biosynthesis of trichothecenes, fumonisin, ochratoxin, aflatoxin and patulin.

## Methods

### Fungal cultures and DNA extraction

A total of forty food-borne fungi posing a health threat in South Africa were obtained from the Agricultural Research Council culture collection (ARC), Pretoria, South Africa and are listed in Table 1. Up to two isolates of each taxon were used depending on availability. Further, eight blind samples were taken at random from the forty fungi to validate the array. Fungal strains were grown on 1.5% malt extract agar at 25°C for 1-2 weeks. Total genomic fungal DNA was extracted following the DNA extraction protocol described by Raeder and Broda [29] and column-purified using the QIAquick PCR Purification Kit (QIAGEN). Total genomic DNA of inoculated maize kernels was isolated by the same protocol.

### Design and identification of unique oligonucleotide probes

For DNA sequencing and subsequent oligonucleotide design, the full ITS regions of all 40 fungi were amplified using universal fungal primers ITS1 (5'-TCCGTAGGTGAACCTGCGG-3') and ITS4 (5'-TCCTCCGCTTATTGATATGC-3') as described by White et al [19]. The PCR amplifications were performed in a 25 μl volume containing 0.4 μM of each universal primer, 1.5 mM MgCl_2_, 0.2 mM of each dNTP, 0.5 U Taq polymerase and 1 × reaction buffer (Bioline) and 8 ng template DNA. The PCR amplification consisted of 35 cycles of denaturation at 94°C for 30 sec, primer annealing at 50°C for 45 sec, and primer extension at 72°C for 1 min; an initial denaturation step of 94°C for 5 min, and a final extension at 72°C for 5 min. Amplicons were then sent for sequencing to Inqaba Biotec (Pretoria, South Africa). Sequenced fragments were aligned using ClustalX [30] in order to identify polymorphisms. For species that showed no significant polymorphisms of the internal transcribed spacer (ITS) regions of the rRNA complex, a different conserved region, namely the elongation factor 1- alpha (EF-1 α) gene, was amplified using primers EF1 and EF2 [31]. The monoplex PCR amplifications were performed in a 20 μl volume containing 0.4 mM of each forward and reverse primer, 0.25 mM of each dNTP, 1× reaction buffer (Bioline), 0.5 U Taq polymerase (Bioline) and 6 ng template DNA. The PCR amplification consisted of 30 cycles of denaturation at 94°C for 30 sec, primer annealing at 57°C for 45 sec, and primer extension at 72°C for 1 min; an initial denaturation of 94°C for 5 min, and a final extension of 72°C for 7 min. The amplified fragments were then column-purified (QIAquick PCR purification Kit, QIAGEN GmbH) and sent for sequencing to Inqaba Biotec (Pretoria, South Africa). The ITS and EF-1 α sequences were then submitted to GenBank [GenBank:FJ864706, GenBank:FJ864709, GenBank:FJ864710, GenBank:FJ864708, GenBank:FJ864711, GenBank:FJ864703, GenBank:FJ864704, GenBank:FJ864705, GenBankFJ864707, and GenBank:FJ864712] and served as targets for the design of multiple probes for each species which are able to discriminate between the forty fungal isolates. The sequences of the conserved regions were aligned using the ClustlX software [30], manual adjustments were made and areas of interspecies variation were identified. These regions were used for the design of genus- and species-specific probes of various lengths (14-25 bases) and within a narrow range of the melting temperature of 56°C (± 5°C). All oligonucleotide probes were designed using the Primer Designer 4 package (Version 4.2, Scientific and Educational Software, Cary, NC). The probe set was then extended by searching public databases (NCBI and EMBL) for genus-or species-specific oligonucleotide probes. The specificity of each oligonucleotide was assessed by conducting BLAST searches and only unique oligonucleotide probes were chosen to be printed onto the array. Probes with similar sequences were then made more stringent by the insertion of LNA (locked nucleic acid) to increase the specificity of each oligonucleotide and to get a set of probes with similar hybridization efficiencies (Table 2). Multiple probes were identified, if possible, for each species or toxin. The probes identified and designed were synthesised by Inqaba Biotech, Pretoria (Pretoria, South Africa).

In addition, the public databases were used to identify toxin-specific probes for genes leading to toxin production for each of the 40 fungi. To test the optimal annealing temperature for array hybridization, monoplex PCR amplifications were carried out for all the probes identified. The PCR amplifications were performed in a 25 μl volume containing 0.4 μM of each oligonucleotide, 1.5 mM MgCl_2_, 0.2 mM of each dNTP, 0.5 U Taq polymerase and 1 × reaction buffer (Bioline) and 5 ng template DNA. The PCR amplification consisted of 30 cycles of denaturation at 94°C for 30 sec, oligonucleotide specific annealing temperatures varying from 55°C to 60°C for 45 sec depending on the primer used, and extension at 72°C for 1 min; an initial denaturation step at 94°C for 5 min, and a final extension step at 72°C for 5 min. Aliquots of amplicons were resolved on 1% agarose gels.

### Array construction

Arrays were constructed from 86 uniquely designed species- and toxin-specific oligonucleotide probes. Equal volumes (10 μl each) of 100 pmol/ml oligonucleotide and 100% DMSO were transferred into a 384-well plate (Amersham PharmaciaBiotech) and stored at -20°C. Sixteen replicates of each oligonucleotide were printed onto Vapour Phase Coated Glass Slides (Amersham Pharmacia Biotech) using a Molecular Dynamics Gen III spotter at the African Centre for Gene Technologies (ACGT) Microarray Facility, University of Pretoria, Pretoria, South Africa http://fabinet.up.ac.za/microarray. Following printing, the slides were allowed to dry overnight at 45-50% relative humidity. Spotted DNA was then bound to the slides by UV cross-linking at 250 mJ and baked at 80°C for 2 h. The DNA internal transcribed spacer oligonucleotides ITS1, ITS3 and ITS4 served as controls for global normalization and were spotted at concentrations of 50 ng/μl, 100 ng/μl, 150 ng/μl and 200 ng/μl onto the array.

### Labeling of target DNA

For target labeling, DNA was extracted from the forty fungi listed in Table 1 using the DNA extraction procedure described before. Extracted DNA was precipitated in 90% ethanol and 0.9 mM NaAc (pH 5.2) to exclude low-molecular-weight fragments. The precipitate was collected by centrifugation at 3,600 g for 30 min. Two micrograms of DNA was labelled with Cy3 or Cy5 by using a Cy™Dye Post-labelling Reactive Dye Pack (GE Healthcare, UK). Each labelling reaction contained DNA diluted in 5 μl 0.2 M Na_2_CO_3 _(pH9) and 2.5 μl Cy5 mono NHS ester 4000 pmol dye resuspended in 12 μl DMSO. The reactions were incubated at room temperature for 90 minutes in the dark. After labeling, the dye coupling reaction was column-purified using the QiaQuick PCR purification (QIAGEN GmbH).

### Hybridization

Vacuum-dried Cy5-labeled target and 0.3 pmol of the Cy3-labeled control probes were resuspended in 40 μl of hybridisation mixture containing 50% formamide (SIGMA), 25% 2× hybridization buffer (Amersham Pharmacia Biotech), and 25% deionized water. This mixture was denatured at 95°C for five minutes and stored on ice for hybridization. The hybridization solution was pipetted onto a glass slide, covered with a cover slip (24 × 60 mm, No.1, Marienfeld, Germany) and inserted into a custom-made hybridization chamber (N.B. Engineering Works, Pretoria, South Africa). The hybridization was performed overnight at 53°C. After hybridization, the slides were washed twice in 2× SSC and 0.2% SDS at 37°C for 6 minutes, once in 0.2× SSC and 0.2% SDS at room temperature for 5 minutes and twice in 0.075× SSC at room temperature for 5 min. The slides were rinsed in de-ionised water for 2 s and dried by centrifugation at 1000 × g for 5 minutes.

### Data acquisition and processing

Oligonucleotide arrays were scanned with a GenePix 4000B scanner (Molecular Dynamics, USA). The mean pixel intensity of each array that resulted from the individual hybridizations was quantified with the Array Vision 6.0 software (Imaging Research Inc., Molecular Dynamics, USA). Individual net signal intensities were obtained by subtracting the local background from the raw spot intensity value. Irregular spots were manually flagged for removal. Further data analysis was performed in the Microsoft Excel software (Microsoft, Richmond, Washington). Anomalous spots not detected through manual inspection were flagged for removal, if the signal intensity of a spot varied more than 10% from the mean of the sixteen replicates on each slide. Signal intensities of the sixteen replicates were then averaged and intensity values were normalized across slides by global regression on the spot intensity data of the internal transcribed spacer oligonucleotides ITS1, ITS3 and ITS4, which were used as a reference for normalization of all spot intensity data (reference design). The net signal intensity of each spot was divided by the median signal intensity of the sixteen replicates and spots with an SNR ((Signal median - Background median) × Standard deviation Background) value below the median were removed from the analysis [32]. Each spot was then either assigned a 1 (present, SNR>/= 3.0) or a 0 (absent, SNR<3.0) according to the median SNR value. The probes with the highest SNR value were considered to be the best target-probe match.

The data discussed above has been deposited at NCBI Gene Expression Omnibus (GEO) [33] and is accessible through GEO series accession number GSE19227.

### Reproducibility of the array

The reproducibility of the array was tested using fungal DNA that was independently extracted from eight blind fungal samples obtained from the Forestry and Agricultural Biotechnology Institute, Pretoria. Fungal DNA was labeled and hybridized to the diagnostic chip. For each hybridization experiment, one technical replicate (using independent labeling reactions) was performed, each replication consisting of a reverse labelling experiment. Data analysis was done as described above and binary scores were obtained. Signal intensity values of replicate hybridizations were plotted against each other in Microsoft Excel to verify that the independent fungal samples showed the same scoring pattern. The results were also compared in each case to the identity obtained for the same culture grown by standard laboratory procedures. In addition, the probes positively identified were used for PCR amplification of the eight samples and the results obtained for the array were confirmed with the PCR product amplified from the same sample. The BLAST program was used to obtain the identities of the amplicons. The same procedure was followed for the mycotoxin biosynthesis genes.

## Abbreviations

DNA: deoxyribonucleic acid; EF-1 α: elongation factor 1-alpha; ITS: internal transcribed spacer; LNA: locked nucleic acids; PCR: polymerase chain reaction; SNP: single nucleotide polymorphisms; ssu rRNA: small subunit ribosomal RNA.

## Authors' contributions

SL: conceived the study, designed the experiment, microarray study, statistical analysis and drafted the manuscript. EB: participated in the study co-ordination and helped to draft the manuscript. Both authors read and approved the final manuscript.
